# Plurality of Fœtuses in the Mare

**Published:** 1830

**Authors:** 


2. *Plurality of Fœtuses in the Mare.*—It is well known that duplicate births from animals of this genus are very rare, and that three are almost never produced. Dr. W. Lindsey has informed us of an instance of the latter kind. Two of the colts were nearly of the usual size, the other was destitute of hair and not larger than a cat, but in good preservation, and possessing distinctly the lineaments of the horse. The parent animal died, probably from protracted parturition, arising from the deformity of one of the colts. Dr. L. was not acquainted with the circumstances, sufficiently early to enable him to investigate the subject, so completely as he wished ; but he concludes very justly, in our estimation, that this case furnishes a very strong objection to the cases brought forward in favor of the doctrine of superfœtation. He is himself able to vouch for the fact that, in this instance, there was no opportunity for superfœtation, and indeed the well known habits of the animal would forbid the idea. The fœtus, from its size and appearance, must have died at an early period of gestation, and consequently, the gravid uterus, while the process of gestation is still going on, must sometimes have the power of arresting the progress of putrefaction in a fœtus, that may accidently die during the early months of pregnancy. This principle, if established, may prove of some importance, by affording a solution to facts that have hitherto greatly puzzled the profession.

The case was further remarkable as an instance of protracted gestation. Linneus has given 290 days as the period of gestation in this species. In the present instance, it was protracted to 355 days—65 days beyond the usual term. F.

